# Supplementary material for: The Two Sweet Sides of Janus Lectin Drive Crosslinking of Liposomes to Cancer Cells and Material Uptake
Source: Toxins (Basel). 2021 Nov 9;13(11):792. doi: 10.3390/toxins13110792 (PMC8620536; doi:10.3390/toxins13110792)
Supplement: Supplementary file 1 [file toxins-13-00792-s001.zip › toxins-1420041-supplementary figures.zip!/toxins-1420041-supplementary figures.pdf]

toxins

MDPI

Supplementary Materials: The Two Sweet Sides of Janus Lectin Drive Crosslinking of Liposomes to Cancer Cells and Material Uptake

Lina Siukstaite, Francesca Rosato, Anna Mitrovic, Peter Fritz Müller, Katharina Kraus, Simona Notova, Anne Imberty and Winfried Römer

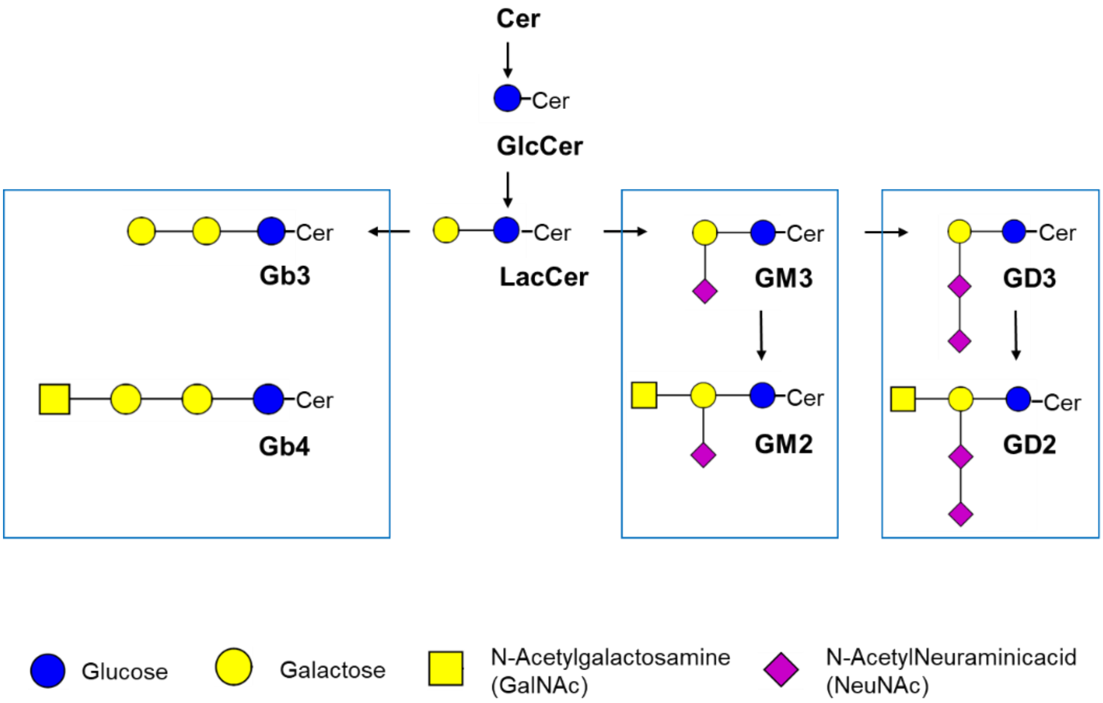

Figure S1. Examples of tumor-associated carbohydrate antigens. Schematic representation of glycolipids found in several malignant tumors.

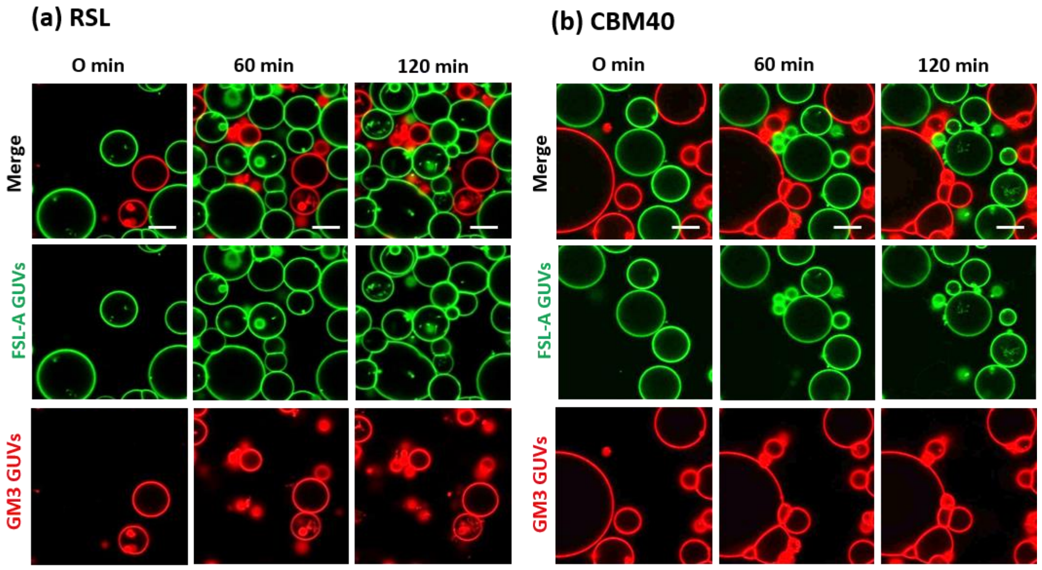

Figure S2. Non-chimeric components of FS-Janus lectin - trimeric RSL and dimeric di-CBM40 do not crosslink GUVs containing different glycospecies. (**a**) Trimeric RSL (200 nM) does not crosslink FSL-A- and GM3-containing GUVs. (**b**) Dimeric di-CBM40 (200 nM) does not crosslink GM3-GUVs with FSL-A-containing GUVs. GM3-GUVs (red color; labeled with the fluorescent lipid DOPE-Atto 647N) and FSL-A-GUVs (green color; labeled with the fluorescent lipid DOPE-Atto 488) were incubated with 200 nM of either RSL or CBM40 (unlabeled) at room temperature and were monitored for 120 min using confocal microscopy. The scale bars represent 10 μm.

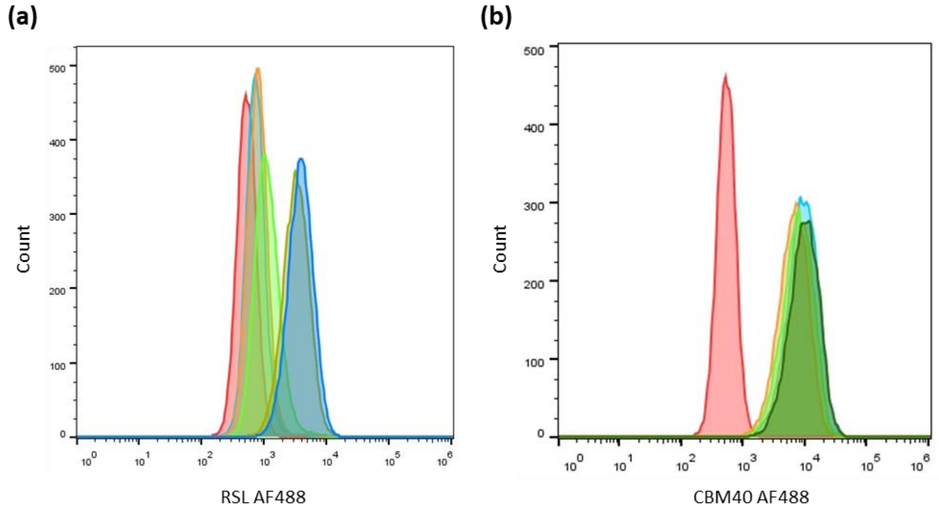

Figure S3. Binding of trimeric RSL and dimeric CBM40 to H1299 in presence of soluble sugars. Flow cytometry analysis of gated living H1299 cells incubated for 30 min at 4 °C with RSL and CBM40 (a, b). (**a**) Histogram of fluorescence intensity of H1299 cells stimulated with 0.2 μM of RSL AF488 pre-incubated with increasing concentrations of soluble L-fucose for 30 min at RT (red: negative control, light blue: RSL + 100 mM L-fucose, orange: RSL + 50 mM L-fucose, light green: RSL + 25 mM L-fucose, green: RSL + 10 mM L-fucose, blue: RSL, untreated). In presence of 100 mM L-fucose, the binding of RSL to H1299 is remarkably reduced and nearly to zero. The 25, 50 and 100 mM L-fucose concentrations were used to saturate FS-Janus fucose-binding sites. (**b**) Histogram of fluorescence intensity of H1299 cells stimulated with 42 nM of CBM40 AF488 pre-incubated with increasing concentrations of 3′-sialyllactose (3′-SL) for 30 min at RT (red: negative control, light blue: CBM40 + 50 mM 3′-SL, orange: CBM40 + 25 mM 3′-SL, light green: CBM40 + 10 mM 3′-SL, green: CBM40, untreated). A total blocking of CBM40 binding to H1299 cells was not achieved in the reported range of inhibitor concentrations. Higher concentrations of 3′-sialyllactose were not sufficient to fully block protein binding to cell surface (not shown). The 25 and 50 mM 3′-sialyllactose concentrations were used to saturate FS-Janus sialic acid-binding sites.

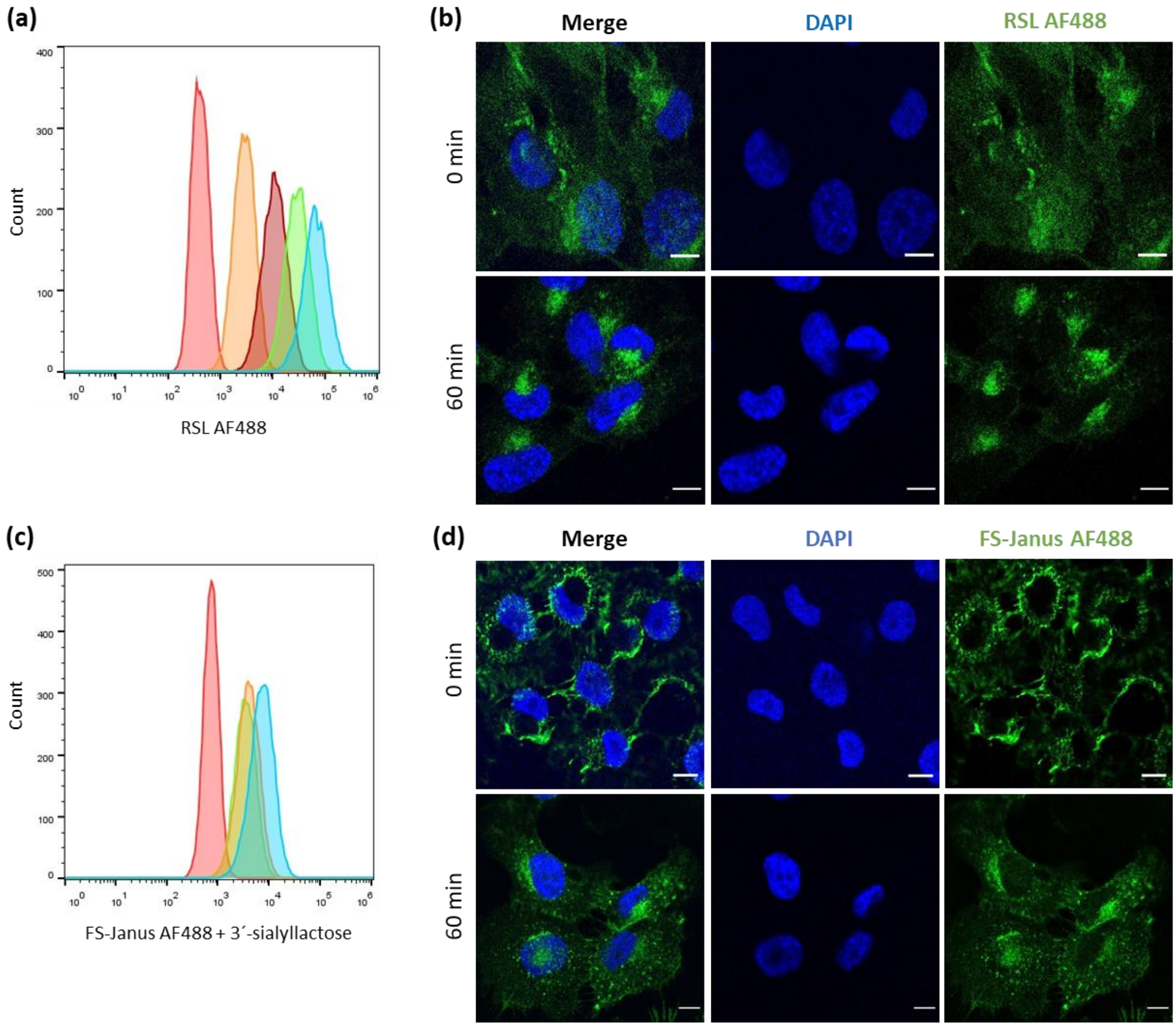

Figure S4. Dose-dependent binding and intracellular uptake of RSL and FS-Janus lectin treated with 3′-sialyllactose in H1299 lung epithelial cells. Flow cytometry analysis of gated living H1299 cells incubated for 30 min at 4°C with RSL (**a**) and FS-Janus lectin + 3′-SL (**c**), confocal imaging of RSL (green color) binding (0 min) and cellular uptake (after 60 min of stimulation) (**b**) and confocal imaging of FS-Janus lectin + 3′-SL at 0 min and after internalization (60 min) (**d**). (**a**) Histogram of fluorescence intensity of H1299 cells stimulated with different concentrations of RSL AF488 (red: negative control, orange: 51 nM, dark red: 0.2 µM, green: 0.5 µM, blue: 10 µM). (**b**) Confocal imaging of fluorescently labelled RSL (green color) incubated with H1299 cells at different time points. Cells were pre-incubated with RSL AF488 (0.62 µM) for 30 min on ice. Subsequently, the lectin solution was washed away and replaced by complete medium, and cells were incubated at 37 °C for 60 min. RSL is internalized within 60 min and accumulates in the perinuclear area. Nuclei were counterstained by DAPI. Scale bars represent 10 µm. (**c**) Histogram of fluorescence intensity of H1299 cells stimulated with FS-Janus AF488 incubated with different concentrations of soluble 3′-sialyllactose for 30 min at RT, to saturate the CBM40 carbohydrate-binding sites (red: negative control, green: FS-Janus lectin 32 nM + 50 mM 3′-SL, orange: FS-Janus lectin 32 nM + 25 mM 3′-SL, blue: FS-Janus lectin 32 nM).). (**d**) Confocal imaging of fluorescently labeled FS-Janus lectin (green color) incubated with H1299 cells at different time points. FS-Janus AF488 (150 nM) was pre-incubated with soluble 3′-sialyllactose (100 mM) for 30 min at RT, and the solution was added to cells. Cells were treated as described in (**b**). In presence of 3′-sialyllactose, FS-Janus lectin is internalized within 60 min and accumulates in the perinuclear region of H1299. Nuclei were counterstained by DAPI. Scale bars represent 10 µm.

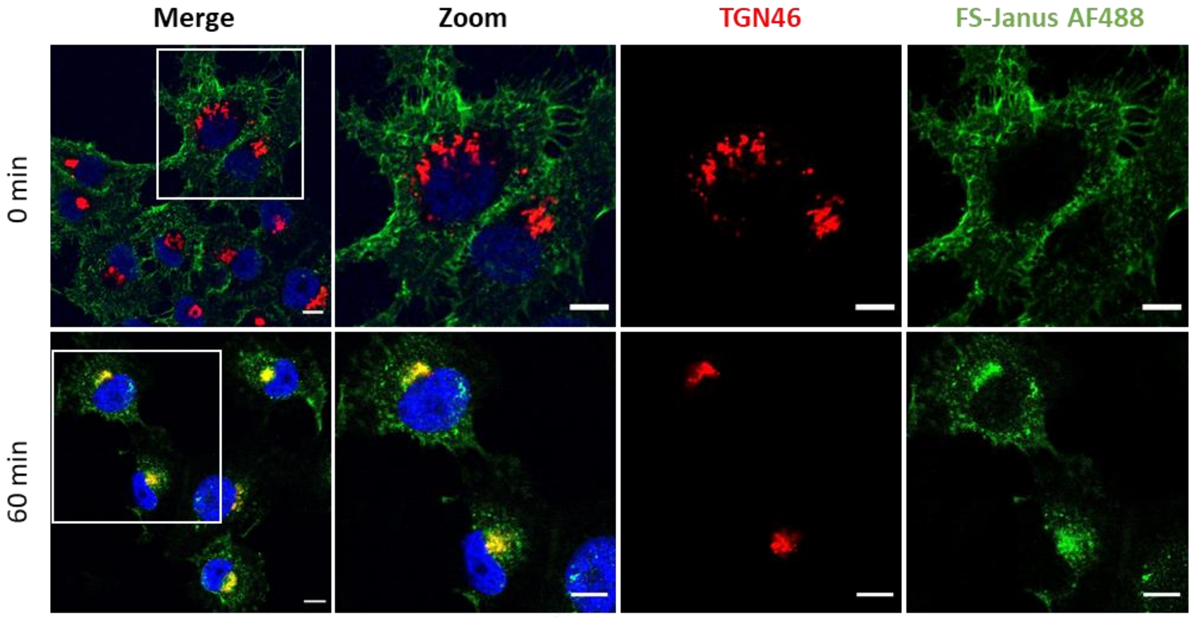

Figure S5. The FS-Janus lectin accumulates at the perinuclear area of H1299. Fluorescence co-localization studies of FS-Janus lectin (green) and *trans*-Golgi network marker TGN46 (red) after 60 min of lectin stimulation. Cells were pre-incubated with FS-Janus AF488 (150 nM) for 30 min on ice. Subsequently, the lectin solution was washed away and replaced by complete medium, and cells were incubated at 37 °C for 60 min. FS-Janus is internalized within 60 min and accumulates at the perinuclear area. Fixed cells were incubated with primary antibody (1:100) for 1 h at RT. After three washes, cells were stained with fluorescently labelled secondary antibody (1:200) for 30 min at RT in the dark. Nuclei were counterstained by DAPI. Scale bars represent 10 µm.

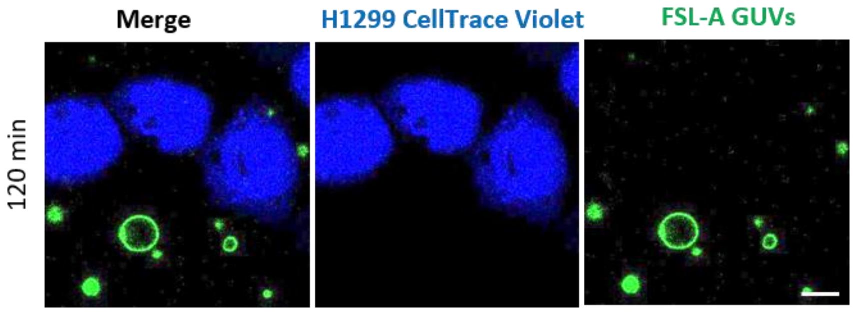

Figure S6. Control experiment of FSL-A GUVs and H1299 cells, co-incubated together without FS-Janus lectin. After 120 minutes of incubation, FSL-A GUVs do not interact visibly with H1299 cells. The GUVs (green color) are labelled with the fluorescent lipid DOPE-Atto488, the H1299 cells (in blue color) are partially stained with CellTrace™ Violet. Scale bars represent 10 µm.

Supplementary Videos

Video S1. FS-Janus lectin triggers crosslinking and lipid exchange between GM3- (red color; labelled with the fluorescent lipid DOPE-Atto 647N) and blood group A trisaccharide- (green color; labelled with the fluorescent lipid DOPE-Atto 488) functionalized liposomes. The crosslinking between GUVs starts immediately after the addition of FS-Janus lectin (200 nM, unlabelled), and lipid exchange becomes apparent during 2 hours of incubation. The scale bar represents 10 µm.

Video S2. Liposome dynamics without the addition of FS-Janus lectin. The GM3- (red color; labelled with the fluorescent lipid DOPE-Atto 647N) and blood group A trisaccharide- (green color; labelled with the fluorescent lipid DOPE-Atto 488) functionalized liposomes do not crosslink and keep their round shape over the entire incubation time. The scale bar represents 10 µm.

Video S3. Crosslinking experiment of blood group A trisaccharide-decorated GUVs and H1299 cells without the addition of FS-Janus lectin. The GUVs (red color) are labelled with the fluorescent lipid DOPE-Atto647N, the H1299 cells (in blue color) are partially stained with CellTrace™ Violet. Live-cell imaging experiments were performed at 37°C by using confocal microscopy and were recorded for 120 min. Scale bar represent 10 µm.

Video S4. Crosslinking of blood group A trisaccharide-decorated GUVs and H1299 cells, which is mediated by FS-Janus lectin (200 nM). The GUVs (red color) are labelled with the fluorescent lipid DOPE-Atto647N, the H1299 cells (in blue color) are partially stained with CellTrace™ Violet and FS-Janus lectin (green color) is labelled with AF488. FS-Janus lectin is enriched at the interfaces. Live-cell imaging experiments were performed at 37°C by using confocal microscopy and were recorded for 120 min. Scale bar represents 10 µm.

Video S5. Kinetics of the cellular uptake and perinuclear co-accumulation of liposomal lipids and FS-Janus lectin. H1299 cells (blue color; partially stained with CellTrace™ Violet) were incubated over in total of three hours in a live-imaging setting with 200 nM FS-Janus lectin (red color, labelled with AF647) and blood group A trisaccharide-decorated GUVs (green color; labeled with DOPE-Atto 488) at 37°C. Scale bar represents 10 µm.

Video S6. Full uptake of blood group A-decorated liposome into H1299 cells. Live-cell imaging experiments were performed at 37°C by using confocal microscopy, and the kinetics of uptake were recorded for 120 minutes. FS-Janus lectin (200 nM, red color; labelled with AF647) triggers the internalization of complete liposomes (green color; labelled with DOPE-Atto 488) into H1299 cells (blue color; partially stained with CellTrace™ Violet). Inside the cell, the liposome remains covered by FS-Janus lectin. Scale bar represents 10 µm.

Video S7. An internalized blood group A trisaccharide-decorated GUV undergoes deformations, shrinkage, and bursts inside H1299 cells. Live-cell imaging experiments were performed at 37°C by using confocal microscopy over 120 minutes. FS-Janus lectin (500 nM, red color; labelled with AF647) triggered the internalization of complete liposomes (green color; labelled with DOPE-Atto 488) into H1299 cells (blue color; partially stained with CellTrace™ Violet). Once internalized, the selected GUV got deformed, reduced its size and bursted. Scale bar represents 10 µm.
